# Effect of pathology type and severity on the distribution of MRI signal intensities within the degenerated nucleus pulposus: application to idiopathic scoliosis and spondylolisthesis

**DOI:** 10.1186/1471-2474-11-189

**Published:** 2010-08-26

**Authors:** Delphine Périé, Daniel Curnier

**Affiliations:** 1 Department of Mechanical Engineering, Ecole Polytechnique, Montréal, QC, Canada; 2 Research Center, CHU Sainte Justine, Montréal, QC, Canada; 3 Department of Kinesiology, Université de Montréal, Montréal, QC, Canada

## Abstract

**Background:**

Disc degeneration is characterized by a loss of cellularity, degradation of the extracellular matrix, and, as a result, morphological changes and biomechanical alterations. We hypothesized that the distribution of the MR signal intensity within the nucleus zone of the intervertebral disc was modified according to the pathology and the severity of the pathology. The objective of this study was to propose new parameters characterizing the distribution of the signal intensity within the nucleus zone of lumbar intervertebral discs, and to quantify these changes in patients suffering from spondylolisthesis or idiopathic scoliosis.

**Methods:**

A retrospective study had been performed on T2-weighted MR images of twenty nine patients suffering from spondylolisthesis and/or scoliosis. The high intensity zone of the nucleus pulposus was semi-automatically detected. The distance "DX" between the center weighted by the signal intensity and the geometrical center was quantified. The sum of the signal intensity on the axis perpendicular to the longitudinal axis of the disc was plotted for each position of the longitudinal axis allowing defining the maximum sum "SM" and its position "PSM".

**Results:**

"SM" was clearly higher and "PSM" was more shifted for scoliosis than for spondylolisthesis. A two-way analysis of variance showed that the differences observed on "DX" were not attributed to the pathology nor its severity, the differences observed on "SM" were attributed to the pathology but not to its severity, and the differences observed on "PSM" were attributed to both the pathology and its severity.

**Conclusions:**

The technique proposed in this study showed significant differences in the distribution of the MR signal intensity within the nucleus zone of intervertebral discs due to the pathology and its severity. The dependence of the "PSM" parameter to the severity of the pathology suggests this parameter as a predictive factor of the pathology progression. This new technique should be useful for the early diagnosis of intervertebral disc pathologies as it highlights abnormal patterns in the MRI signal for low severity of the pathology.

## Background

The nucleus pulposus is a viscous gel that is approximately centrally located within the intervertebral disc. The proteoglycans of the nucleus osmotically exert a swelling pressure which enables it to support spinal compressive loads. The pressurized nucleus also creates tensile stresses within the collagen fibers of the annulus and ligamentous structures surrounding the nucleus.

Intervertebral disc degeneration is characterized by a loss of cellularity, degradation of the extracellular matrix, and, as a result, morphological changes and alterations in biomechanical properties. Secondary changes from redistribution of tissue stress include fibrocartilage production, with disorganization of the annular architecture and increases in type-II collagen. The nucleus pulposus becomes more consolidated and fibrous, and is less clearly demarcated from the annulus pulposus. Early degenerative changes occur in the nucleus pulposus [1,2].

Adolescent idiopathic scoliosis is a complex three-dimensional disorder of the spine involving deviations in the frontal plane, modifications of the sagittal profile, rotations in the transverse plane, and alterations of the rib cage. Local structural deformities develop concurrently in pedicles, spinous and transverse processes, vertebral bodies, and intervertebral discs. With idiopathic scoliosis, the intervertebral discs become wedged and narrowed, due in part to altered biomechanical environment [3]. Biochemical changes were also observed, such as lower glycosaminoglycan content, increased collagen content or increased total protein content in the nucleus pulposus or in the convex side of scoliotic intervertebral discs [4-8].

Spondylolisthesis is an abnormal anterior translation of one vertebra on another in the sagittal plane and is due to the degeneration of the supporting structures of the functional spinal unit: the intervertebral disc, intervening muscles and ligaments, capsulae, and facet joints. MacMahon et al. [9] found a significant upward vertical and lateral disc displacement to the exit foramen in patients with spondylolisthesis.

Over other medical imaging techniques, magnetic resonance imaging (MRI) has the advantage of using non ionising radiation, and early MRI work on intervertebral discs in patients with back pain consisted in the detection of degenerative disc abnormalities. Diminished signal intensity in the disc and evidence of radial tears in the annulus fibrosus are highly associated with positive symptoms on discography.

Changes in the intervertebral disc height between morning and evening measurements [10], expansion of the disc area during overnight or long bed rest [11], or volume increases after removing a highly compressive load [12] were quantified from MRI images. For early idiopathic scoliosis, no significant volume variation was detected on the intervertebral discs [13] whereas the postoperative volumes increased significantly for nucleus, disc, and nucleus-disc ratio for patients with scoliosis treated by surgery [14].

The displacements of the intervertebral disc components under various movements of the spine were also measured from MRI images, highlighting significant correlations between the nucleus zone migration and the flexion-extension movements of the spine [15-17]. Creep displacements were also measured in lumbar intervertebral discs from loaded and unloaded MRI scans [18]. For idiopathic scoliosis, correlations between nucleus zone migration and intervertebral disc wedging [19] and significant differences on the disc migration at the apex of the curve [13] were observed. Moreover, after surgical treatment, this disc migration could be conditioned by the location of the surgical instrumentation [14].

We believe that the pattern of the MRI signal within the intervertebral disc is a predictive factor of the progression of its degenerescence. The validation of this hypothesis will be relevant in the future for clinical application. Thus our global objective is to develop powerful image analysis tools for the early diagnosis of intervertebral disc pathology and the prediction of its evolution. This paper presents the first step toward this goal. We hypothesized that the distribution of the MR signal intensity within the nucleus zone of the intervertebral disc is modified according to the pathology and the severity of the pathology. The objective of this study was to propose new parameters characterizing the distribution of the signal intensity within the nucleus zone of lumbar intervertebral discs, and to quantify these changes in patients suffering from spondylolisthesis or idiopathic scoliosis.

## Methods

A retrospective study has been performed on the MR images of patients suffering from spondylolisthesis or idiopathic scoliosis. They all signed an informed consent validated by the ethic comitee of the CHU Sainte Justine (Montreal, Canada), in agreement with the Helsinki Declaration.

They underwent an MRI acquisition on a 1.5 T system (Sonata system, Siemens Healthcare, Erlangen, Germany) using the spine coil to maximise the signal, composed of two sagittal and one axial turbo spin-echo sequences. We were interested only in the T2-weighted sagittal slices of the lumbar spine (slice thickness of 4 mm, matrix of 512 × 512 and field of view varying from 320 × 320 mm^2 ^to 400 × 400 mm^2^), in which the nucleus zone can be differentiated from the annulus zone using the segmentation of the high signal intensity zone.

The inclusion criteria were patients suffering from spondylolisthesis or idiopathic scoliosis, from 5 to 18 years old, MR acquisition parameters TR/TE of 3200/124. The exclusion criteria were incomplete patient file, and TR/TE different from 3200/124. A total of fourteen adolescent patients with spondylolisthesis (age 14.7 ± 2.9, 10 females and 4 males) and fifteen adolescent patients with idiopathic scoliosis (age 13.6 ± 2.7, 9 females and 6 males) have been included.

Three levels of spondylolisthesis were defined from the Meyerding classification: 1) level 1 for grades I and II; 2) level 2 for grades III and IV; 3) Level 3 for grade V. Three levels of idiopathic scoliosis were defined from the clinical measure of the Cobb angle, performed by the surgeon on the radiography: 1) level 1 for a Cobb angle inferior to 20°; 2) level 2 for a Cobb angle between 21° and 40°; 3) level 3 for a Cobb angle superior to 41°.

**Figure 1:**
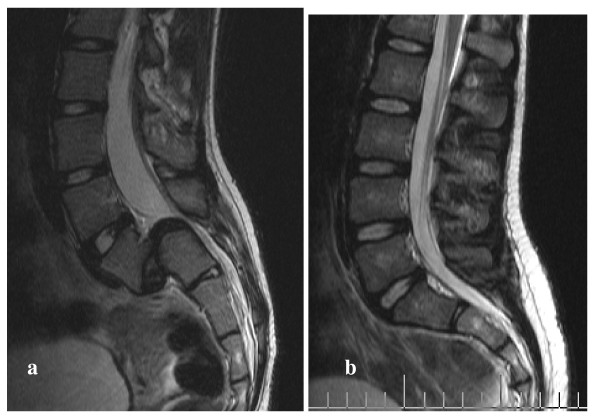
**T2-weighted MR images of lumbar spine**. T2-weighted sagittal MR images of a patient (female, 13 years old), suffering from spondylolisthesis (a) and a patient (female, 14 years old) suffering from idiopathic scoliosis (b)

**Figure 2:**
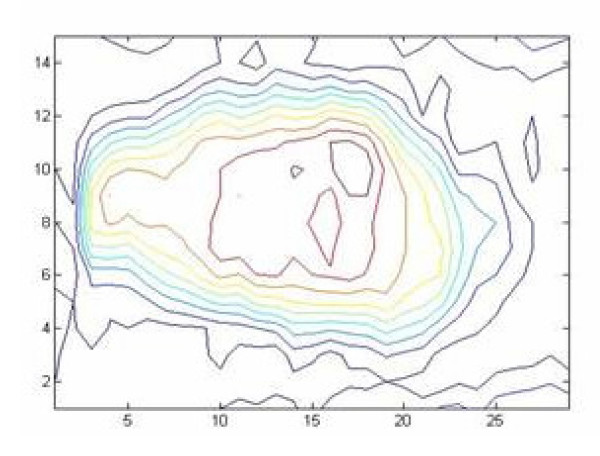
**High intensity zone segmentation**. Segmentation of the high intensity zone of the disc using Matlab functions.

Each lumbar intervertebral disc, from L5/S1 to T12/L1, was classified according to the Thompson scale, and analysed using the most central MR slice (Figure 1). The high intensity zone of the nucleus pulposus was semi-automatically detected (Figure 2, Matlab, Mathworks, Natick, MA). This task was done by experienced observers (2 Biomedical Engineers) with Matlab software and segmentation tools.

The geometrical center and the height "H" (mm) of the nucleus were quantified. A center weighted by the signal intensity within the nucleus was determined according to equation 1, where Xi is the coordinate of each pixel, and Si the signal intensity of each pixel. The distance between the weighted center and the geometrical center on the longitudinal axis of the disc was called "DX" (mm).

(1) $$X=\frac{\sum XiSi}{\sum Si}$$

**Figure 3:**
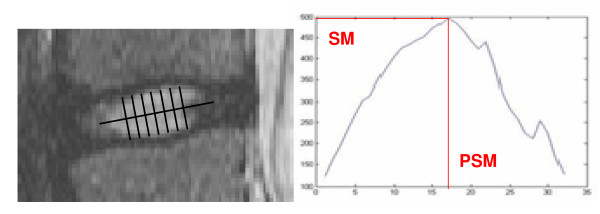
**Calculus method of proposed parameters**. Calculus method of the maximum sum of the signal intensity "SM" and its position "PSM" on the disc longitudinal axis.

The sum of the signal intensity on the perpendicular axis to the longitudinal axis of the disc was computed for each pixel of the longitudinal axis (Figure 3). The maximum sum was called "SM", and its position on the longitudinal axis "PSM" (mm).

The post-processing takes about 5 minutes, including the image selection, the outline of the nucleus using Matlab and finally the running of the Matlab program that compute the described parameters.

### Reproducibility of the method

The inter and intra operator reproducibility was tested by the same two observers who realised the analysis three times on a same patient. First of all, they chose the MR image to be analysed for each of the three discs L4/L5, L3/L4 and L2/L3. For each disc, they detected the nucleus zone and quantified the parameters "DX", "H", "SM" and "PSM". The standard deviation was evaluated for the 6 repetitions of the 2 observators, and for each of the three repetitions of each observator.

### Statistical tests

Two-way analysis of variances (Sigma Stat^®^, Jandel Scientific, San Rafael, CA) was used to analyse the influence of the pathology and its level on the distribution of the MR signal intensity within the nucleus zone of the intervertebral disc. The pathology (scoliosis vs spondylolisthesis) was the first factor, and the severity of pathology (level 1 to 3) was the second factor.

## Results

The inter and intra-observator reproducibility was inferior to 0.02 mm for "DX", "H" or "PSM", and less than 0.1% for "SM".

**Figure 4:**
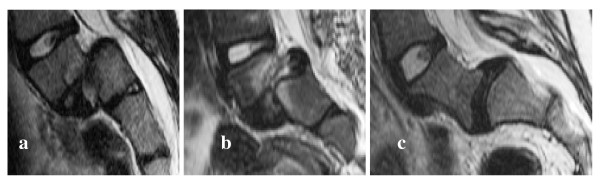
**T2-weighted MR images of intervertebral discs L5/S1 of patients suffering from spondylolisthesis**. The disc is cut in two parts (a and b) or the high intensity zone representing the nucleus does not exist (c). The patients were all females, 15 (a), 13 (b) and 16 (c) years old.

The L5/S1 disc in spondylolisthesis was largely degenerated from grade II to V, with low signal intensity and absence of high intensity zone (Figure 4), preventing its analysis. Thus only the intervertebral discs L4/L5, L3/L4 and L2/L3 were analysed for spondylolisthesis. Moreover, patients' files reported pathologies associated to the spondylolisthesis, such as scoliosis, bone abnormalities within the vertebral endplates, diffuse disc buckling, or intervertebral degeneration (height loss for example). For idiopathic scoliosis, all intervertebral discs were less degenerated with a higher MR signal intensity than for spondylolisthesis.

Table 1 presents the mean and standard error of estimate for each of the proposed parameters for each level of pathology. The maximal sum of signal intensity "SM" is clearly higher and its position "PSM" is more shifted for scoliosis than for spondylolisthesis. This shift decreases with increasing the pathology severity. The distance between the weighted center and the geometrical center "DX" decreases with increasing the scoliosis severity, whereas it increases with increasing the spondylolisthesis severity.

**Table 1:** Results on the proposed parameters

	Spondylolisthesis			Scoliosis		
**Level**	**1**	**2**	**3**	**1**	**2**	**3**

**DX (mm)**	0.39 ± 0.22	1.15 ± 0.25	1.10 ± 0.56	1.60 ± 0.18	0.80 ± 0.20	0.64 ± 0.35

**SM**	765 ± 76	873 ± 94	512 ± 210	1139 ± 70	1241 ± 74	1171 ± 129

**PSM (mm)**	10.18 ± 0.55	9.44 ± 0.69	6.38 ± 1.53	15.08 ± 0.51	13.97 ± 0.54	12.27 ± 0.94

**H (mm)**	8.92 ± 0.31	8.10 ± 0.38	6.88 ± 0.86	7.85 ± 0.28	7.61 ± 0.30	7.46 ± 0.53

Mean ± SEM of the proposed parameters, for each pathology and level of pathology.

The results of the analysis of variance (Table 2) showed that the differences observed on "DX" were not attributed to the pathology (p = 0.6) nor its severity (p = 0.9), the differences observed on "SM" were attributed to the pathology (p < 0.001) but not to its severity (p = 0.2), the differences observed on "PSM" were attributed to both the pathology (p < 0.001) and its severity (p = 0.003), and the differences observed on "H" were attributed to the severity of the pathology (p = 0.05) but not to the pathology (p = 0.4).

**Table 2:** Two-way analysis of variance results.

Data	p for pathology	p for level
**DX**	0.6	0.9

**SM**	< 0.001	0.2

**PSM**	< 0.001	0.003

**H**	0.4	0.05

## Discussion

A retrospective study had been performed on MR images of patients suffering from spondylolisthesis and scoliosis. New parameters allowing analysing the distribution of the signal intensity within the nucleus zone of the lumbar intervertebral disc were defined and quantified.

The reproducibility analysis showed very low standard deviations on the repeated measures, suggesting a high reproducibility of the developped methodology. The only possible source of dispersion was the manual choice of the nucleus zone outline in the segmentation tool of Matlab. For each observatory, the training performed for the first detection entailed the same choice for the following detections. For two different operators, the outlines proposed by the software entailed an evident choice. Because of our reproducibility results, it was considerated unnecessary to perform a more detailed analysis.

The higher maximal sum of signal intensities for scoliosis than for spondylolisthesis reflects higher disc degeneration for spondylolisthesis than for scoliosis. The general decrease observed in the signal intensity within the intervertebral disc (not the "SM" parameter limited to the nucleus) with increasing the severity of scoliosis could be related to the lower glycosaminoglycan content and increased collagen content in the nucleus pulposus of scoliotic discs observed by Pedrini et al [5] and Zaleske et al. [6]. The decrease of "PSM" values for scoliosis reflects a shift of the nucleus zone into the convex side of the scoliotic curve and could be related to the biochemical changes observed in the intervertebral discs with scoliosis. Bushell et al. [4] showed that the concave side of the scoliotic curve had lower levels of collagen than tissues from the convex side of the curve. Antoniou et al [7] found higher collagen Type II synthetic levels and increased total protein content with no matrix turnover in the convex side of scoliotic intervertebral discs.

This study revealed modifications in the distribution of the MR signal intensity within the nucleus zone of the lumbar intervertebral disc. However, our hypothesis was only partially verified. The variance analyses suggest that "DX" is not sensitive to the variations that occur with the pathology. This parameter averages the modifications that could occur in the distribution of the signal intensities. However, for idiopathic scoliosis, the analysis was performed in the sagittal plane, wich is not the maximum plane of deformation. For spondylolisthesis, only the adjacent discs to the pathologic disc were analysed. "DX" is not a sensitive parameter for these adjacent discs, but the question remains for the highly degenerated L5/S1 disc. The variance analyses suggest that "PSM" is sensitive to the variations that appear with the pathology and its severity. It is well known that the intervertebral height decreases with disc degeneration, which is illustrated in our results by the sensitivity of "H" to the severity of the pathology (p = 0.05). Chen et al. [20] measured lower disc height for a group of patients with spondylolisthesis than for a control group, with significant differences between both groups. This is in agreement with our nucleus height "H", which is a parameter slightly different than the disc height measured by Chen et al. We found a decrease of "H" with increasing the severity of spondylolisthesis.

While this 2D analysis showed interesting variations in the distribution of the MR signal intensity within the nucleus zone of the intervertebral disc depending on pathology and severity, spondylolisthesis and scoliosis are 3D complex deformations. Future developments will include a 3D analysis, an optimized filtering of the images, and iso-intensity volume krigging.

In the literature, mean values over specific region of interest (ROI) are often used to quantify MR parameters within the intervertebral disc submitted to various conditions (enzyme digestion, loading...). This ROI can be difined by the full intervertebral disc [10,21], the mean half signal intensity of the disc [11], the high intensity zone of the nucleus pulposus [22-24], three squares (located on the anterior annulus fibrosus, nucleus pulposus, and posterior annulus fibrosus, [25]), six squares along the longitudinal axis of the disc [26], small polygonal shapes aligned within the center of the NP or localized in the anterior and posterior annulus fibrosus [27], a central round [28], 5-mm-diameter circular regions of interest located in the center of the high intensity zone of the nucleus [29] or else nine circles (one positioned at the center of the nucleus, four positioned at the rim of the discs and four positioned in the intermediate area, [30]). For all these authors, the distribution of the MR parameters within the nucleus zone or intervertebral disc is not quantitatively analysed. However, Vaga et al. [31] quantified the glycosaminoglycan distribution within the intervertebral discs of orthopedic patients using Gadolinium-Enhanced T1 parametric image subdivided into 60 sectors. For each sector, a mean value of T1 was computed allowing a qualitative analysis of the distribution. To investigate fluid flow and global permeability from high-resolution MRI, Swider et al. [32] analysed each parametric image using histogram (number of pixels as a function of signal intensity) of square ROIs and binary representations of the same ROIs allowing a quantitative analysis of the distribution of the signal intensity within the tissue. This last technique will be applied in 3D on these patients with spondylolisthesis or idiopathic scoliosis.

## Conclusions

The T2-weighted MRI images did not show qualitative differences in the distribution of the signal intensity within the nucleus zone of these patients with scoliosis or spondylolisthesis. However, the quantitative analysis proposed in this study showed significant differences in the distribution of the MR signal intensity within the nucleus zone of intervertebral discs due to the pathology and its severity. The dependence of the "PSM" parameter to the severity of the pathology suggests this parameter as a predictive factor of the pathology progression, but needs follow-up studies to confirm this hypothesis. This new technique should be useful for the early diagnosis of intervertebral disc pathologies as it highlights abnormal patterns in the MRI signal for low severity of the pathology.

## Competing interests

The authors declare that they have no competing interests.

## Authors' contributions

DP participated in the design of the study, carried out the images analysis and the definition of the new parameters quantifying the distribution of the MRI signal intensity, and drafted the manuscript.

DC participated in the design of the study, performed the statistical analysis and helped to draft the manuscript.

Both authors read and approved the final manuscript.

## Pre-publication history

The pre-publication history for this paper can be accessed here:

http://www.biomedcentral.com/1471-2474/11/189/prepub
